# Breast-Associated Adipocytes Secretome Induce Fatty Acid Uptake and Invasiveness in Breast Cancer Cells via CD36 Independently of Body Mass Index, Menopausal Status and Mammary Density

**DOI:** 10.3390/cancers11122012

**Published:** 2019-12-13

**Authors:** Maurice Zaoui, Mehdi Morel, Nathalie Ferrand, Soraya Fellahi, Jean-Philippe Bastard, Antonin Lamazière, Annette Kragh Larsen, Véronique Béréziat, Michael Atlan, Michèle Sabbah

**Affiliations:** 1 Institut National de la Santé et de la Recherche Médicale (INSERM), Centre National de la Recherche Scientifique (CNRS), UMR_S 938, Centre de Recherche Saint-Antoine, Team Cancer Biology and Therapeutics, Institut Universitaire de Cancérologie, Sorbonne Université, F-75012 Paris, France; maurice.zaoui@inserm.fr (M.Z.); mehdi.morel@inserm.fr (M.M.); nathalie.ferrand@inserm.fr (N.F.); annette.larsen@sorbonne-universite.fr (A.K.L.); 2 Department of Biochemistry and Hormonology, Tenon Hospital, AP-HP, F-75020 Paris, France; soraya.fellahi@aphp.fr (S.F.); jean-philippe.bastard@tnn.aphp.fr (J.-P.B.); 3 Institut National de la Santé et de la Recherche Médicale (INSERM), Centre National de la Recherche Scientifique (CNRS), UMR 70203, Laboratory of Biomolecules, École Normale Supérieure, AP-HP, F-75012 Paris, France; antonin.lamaziere@upmc.fr; 4 Institut National de la Santé et de la Recherche Médicale (INSERM), Centre National de la Recherche Scientifique (CNRS), UMR_S 938, Centre de Recherche Saint-Antoine, Team Genetic and Acquired Lipodystrophies, Institut Hospitalo-Universitaire de Cardiométabolisme et Nutrition, Sorbonne Université, F-75012 Paris, France; veronique.bereziat@inserm.fr; 5 Department of Plastic Surgery, Reconstructive, Aesthetic, Microsurgery and Tissue Regeneration, Tenon Hospital, Institut Universitaire de Cancérologie, AP-HP, F-75020 Paris, France; drmichaelatlan@gmail.com

**Keywords:** breast cancer, adiposity, body mass index, menopausal status, mammary density, fatty acid, CD36

## Abstract

Breast adiposity is correlated with body mass index, menopausal status and mammary density. We here wish to establish how these factors influence the cross-talk between breast adipocytes and normal or malignant breast cells. Adipocyte-derived stem cells (ASCs) were obtained from healthy women and classified into six distinct groups based on body mass index, menopausal status and mammary density. The ASCs were induced to differentiate, and the influence of their conditioned media (ACM) was determined. Unexpectedly, there were no detectable differences in adipogenic differentiation and secretion between the six ASC groups, while their corresponding ACMs had no detectable influence on normal breast cells. In clear contrast, all ACMs profoundly influenced the proliferation, migration and invasiveness of malignant breast cells and increased the number of lipid droplets in their cytoplasm via increased expression of the fatty acid receptor CD36, thereby increasing fatty acid uptake. Importantly, inhibition of CD36 reduced lipid droplet accumulation and attenuated the migration and invasion of the breast cancer cells. These findings suggest that breast-associated adipocytes potentiate the invasiveness of breast cancer cells which, at least in part, is mediated by metabolic reprogramming via CD36-mediated fatty acid uptake.

## 1. Introduction

Breast cancer remains the leading cause of cancer death in women and is increasing worldwide [1]. The increased incidence is not a result of aging, since it is observed for all age groups, suggesting that other risk factors should be considered. Risk factors can be divided into non-modifiable factors, such as hormone treatments, early menarche, nulliparity, late pregnancy and not breastfeeding, or modifiable risk factors, like obesity and low physical activity [2].

Breast cancer has been stratified into different molecular subgroups, that are associated with different prognosis. This has permitted a distinction between Estrogen Receptor (ER) expressing luminal cancers (subdivided into luminal A and B according to progesterone Receptor (PR) expression and Ki-67), HER2-positive cancers (overexpression of HER2) and triple negative cancers (no detectable expression of ER, PR or HER2), which are all associated with different prognoses [3].

During the past decade, abundant evidence has associated obesity with an increased incidence of breast cancer [4,5]. Obesity induces inflammation of the adipose tissue, which will induce an inflammatory state leading to insulin resistance [6,7]. This inflammatory state leads to elevated levels of circulating leptin and proinflammatory cytokines—factors that have previously been implicated in breast cancer progression [8,9,10]. In addition, breast cancer is affected by a high body mass index (BMI) and depends on menopausal status and different breast cancer subtypes [11]. Since AT becomes an important source of estrogen due to the high local expression of aromatase in postmenopausal women [12], obesity increases the risk of postmenopausal women developing hormone receptor-positive breast cancers [13,14,15]. In contrast, recent studies of premenopausal women provide evidence for a relationship between overweight/obesity and an increased risk of triple negative breast cancer, but a lower risk for hormone receptor-positive breast cancer [15,16,17].

A normal mammary gland is constituted of canals and lobules, together with a stromal fraction consisting of fibroblasts, endothelial cells, macrophage, immune cells and adipocytes as major constituents. In breast cancer, the tumour grows in an adipose-rich stroma. [18]. However, the relationship between breast fat and breast cancer risk is not well understood. In this context, mammographic density is of particular interest, since almost one third of breast cancer is thought to be associated with high breast density [19,20,21]. Paradoxically, despite the fact that adiposity is positively associated with breast cancer risk (World Cancer Research Fund, American Cancer Institute for Cancer research, 2010), several studies have reported a negative association between the amount of AT in the breast, reflected by the absolute non-dense area (mainly AT), and breast cancer risk [22,23].

Adipose-derived stem cells (ASCs) are a heterogeneous group of cells within the extracellular matrix of the AT surrounding mature adipocytes [24]. ASCs are present in normal breast AT, and have the ability to differentiate into mature adipocytes in vivo and in vitro [25]. Interestingly, accumulating experimental evidence has shown that ASCs and adipocytes play an active role in breast cancer progression through paracrine signals that are released locally within the tumor micro-environment [8,26]. Given the complexity of the AT-tumor cell cross-talk, which depends on multiple factors, including menopausal status, mammary density and BMI, a major challenge is to determine how breast-associated AT contributes to breast tumor growth and progression.

The objective of this study is to determine if body mass index, menopausal status or mammary density affect the crosstalk between adipocytes and normal or malignant breast cells, and to what degree this could promote the malignant phenotype. Unexpectedly, the influence of the adipocytes on breast cells was independent of BMI, menopausal or mammary density status. None of the different adipocyte-conditioned media (ACM) had any detectable influence on normal breast cells. In marked contrast, all ACMs were able to induce metabolic reprogramming of breast cancer cells by elevated expression of the fatty acid receptor CD36, accompanied by increased invasive potential.

## 2. Results

### 2.1. Adipocyte Differentiation of ASCs as A Function of BMI, Menopausal Status and Mammary Density

**Figure 1:**
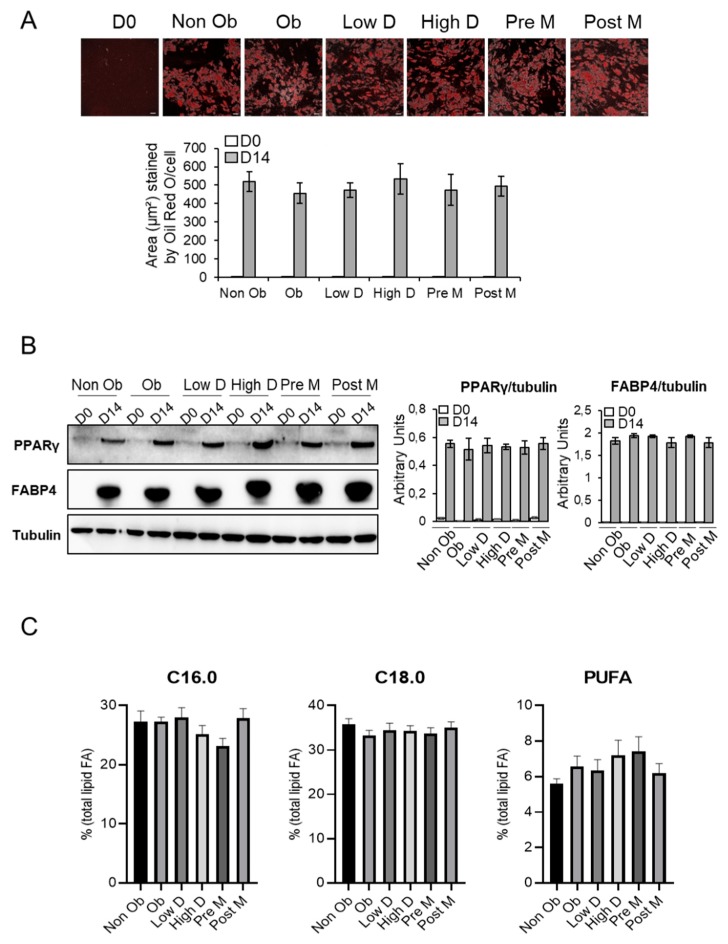
Adipocyte differentiation of adipocyte-derived stem cells (ASCs) populations. Breast ASCs were differentiated into adipocytes for 14 days. (**A**) The differentiation process was monitored by microscopic imaging after Oil Red O-staining (upper panel). Subsequently, Oil Red O-stained areas were quantified by ImageJ analysis. Scale bars, 70 µm. Results are presented as means ± standard error of the mean (SEM) (lower panel). (**B**) Whole cell lysates were extracted at day 0 and 14 of ASCs differentiation and were subjected to immunoblotting with anti-PPARγ and anti-FABP4 antibodies (left panel). Immunoblot signals were quantified by densitometry, and normalized with tubulin. Data are representative of three individual samples from three independent experiments. Results are presented as means ± SEM (right panel). (**C**) Dosage of Free Fatty Acids (FFAs). C16.0, palmitic acid; C18.0, stearic acid; PUFA, polyunsaturated fatty acids. Results are presented as means ± SEM. The uncropped blots and molecular weight markers are shown in Supplementary Materials.

To study the effects of BMI, menopausal status and mammary density on breast adipocyte differentiation and secretion, mammary human adipose-derived stem cells (ASCs) were isolated from 16 different breast tissues obtained from healthy, cancer-free women who underwent mammoplasty. Women were classified into six distinct groups based on body mass index (10 non obese women, having a body mass index (BMI) of <30, and six obese women, having a BMI ≥ 30; mean BMI 25.5 ± 0.84 versus 32.1 ± 0.94 kg/m^2^), menopausal status (seven in pre-menopausal status (Pre-M) and nine in post-menopausal status (Post-M) and mammary density (nine women showing low mammary density (Low D) and seven high mammary density (High D) (Table S1). The ASCs were differentiated in vitro and the serum-free conditioned media (ACM) was collected. The adipogenic capacity of the ASCs was evaluated by Oil-Red-O staining and expression of the adipocyte markers PPARγ and FABP4. As shown in Figure 1A, ASCs from the different groups showed a similar increase in lipid accumulation after 14 days of differentiation.

In agreement with each other, the expression of PPARγ and FABP4 were increased in a similar manner between the different groups (Figure 1B and Figure S1). Furthermore, total lipids fatty acids (FA) profiles were compared between the different groups. No changes were observed in saturated FA C16:0 and C18:0 and polyunsaturated FA (PUFA) levels (Figure 1C and Figure S2).

**Figure 2:**
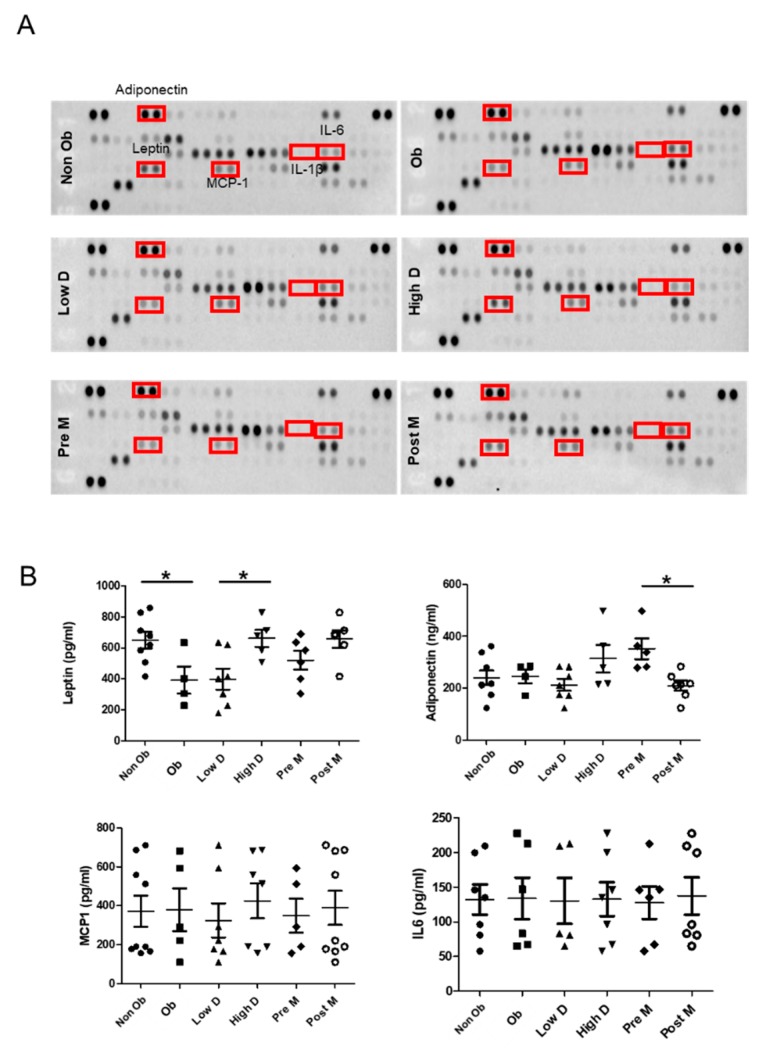
Adipokine expression by different ASCs populations. (**A**) Serum-free conditioned medium from the indicated adipocytes was applied onto human adipokine array membranes. The boxes indicate adipokines further tested by ELISA. (**B**) Scatter plots of leptin, adiponectin, MCP1 and IL6 concentrations in conditioned medium are shown from 16 breast ASCs populations after 14 days of differentiation, as measured by ELISA, * *p* < 0.05.

To further analyze the secreted adipokines, the ACMs were analyzed using a human adipokine array. Overall, there were no major differences between the six different groups (Figure 2A). However, the levels of the pro-inflammatory cytokine IL-6 were higher in the ACM derived from obese individuals compared to what was observed for the ACM derived from non-obese individuals, while the opposite was observed for leptin. Interestingly, leptin levels were also lower in ACM derived from individuals with low mammary density. Surprisingly, we did not detect any noticeable differences in adiponectin, IL1β and MCP1 levels.

To verify these results, ELISA assays were performed. The results confirmed that the concentration of leptin is higher in ACM obtained from non-obese women compared to their obese counterparts, and also lower in women with low mammary density compared to individuals with high mammary density. These findings suggest that high leptin levels in the breast are associated with low adiposity. Moreover, we found an increase in adiponectin in adipocytes from Pre-M compared to Post-M (Figure 2B). The levels of IL6 and MCP1 showed important inter-individual differences in a non-group related manner (Figure 2B). Finally, the levels of IL1b remained undetectable under all conditions).

### 2.2. The Secretome of Breast Adipocytes Increases the Proliferation of Tumor Cells Independently of BMI, Menopausal and Mammary Density Status

**Figure 3:**
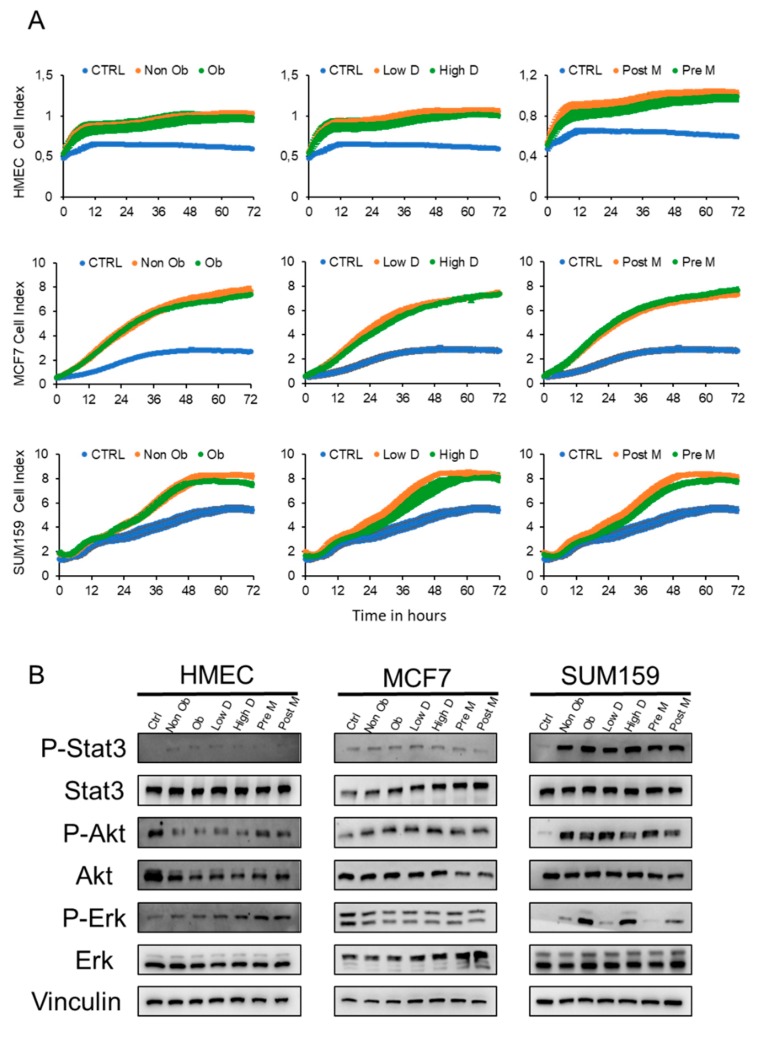
Adipocyte conditioned medium stimulates proliferation of tumor cells independently of BMI, menopausal status and mammary density. (**A**) Graphs representing the proliferation of HMEC, MCF7 and SUM159 cells treated with control medium (CTRL) or conditioned medium from ASCs with the indicated characteristics differentiated into adipocytes. The proliferation rate was assessed by the xCELLIgence system RTCA system. Data are representative of three individual samples from three independent experiments. (**B**) Cell extracts were prepared from HMEC, MCF7 and SUM159 cells treated for 1 h with either control medium or the indicated adipocyte conditioned media (ACM) and characterized by Western blot analysis. The uncropped blots and molecular weight markers are shown in Supplementary Materials.

We then investigated whether the adipocyte secretome (ACM) influences the proliferation of normal breast cells (HMEC) and breast cancer cells, corresponding to the luminal A (MCF7) or the triple negative (SUM159) subtypes. Cells were treated with medium containing 0.25% serum (CTRL) or different ACM supplemented with 0.25% serum and proliferation was measured for 72 h using an Xcelligence system. The results (Figure 3A) show no detectable proliferation of the HMEC cells under low serum conditions (0.25% FCS). In comparison, both MCF-7 and SUM159 cells are able to proliferate, with the proliferative capacity of the SUM159 cells being stronger than that of MCF7. The addition of ACM had minor influence on the proliferation of HMEC cells, whereas the ACM induced a strong increase in the proliferation (3-fold) of MCF-7 cells, compared to a more modest increase for SUM159 cells (1.5-fold). These findings were independent of the type of ACM (Figure 3A and Figure S3).

We then determined which proliferative pathways were induced by the ACM in the different cell lines. No detectable activation of any of the studied signaling pathways was observed for HMEC cells (Figure 3B, Figures S4 and S5). In clear contrast, activation of AKT signaling was observed for both MCF7 and SUM159 cells, whereas a clear activation of the STAT3 and ERK1/2 pathways was also observed for SUM159 cells. Interestingly, ERK1/2 activation was higher in the presence of ACM derived from obese, High D and Post-M individuals, than in the ACM derived from other groups (Figure 3B, Figures S4 and S5). Together, these results show that ACM increases the proliferation of breast tumor cells, but has no detectable influence on normal cells, independent of which type of individual the ACM was derived from.

### 2.3. The Influence of Adipocyte-Derived Conditioned Medium (ACM) on the Migration and Invasion of Breast Cells

**Figure 4:**
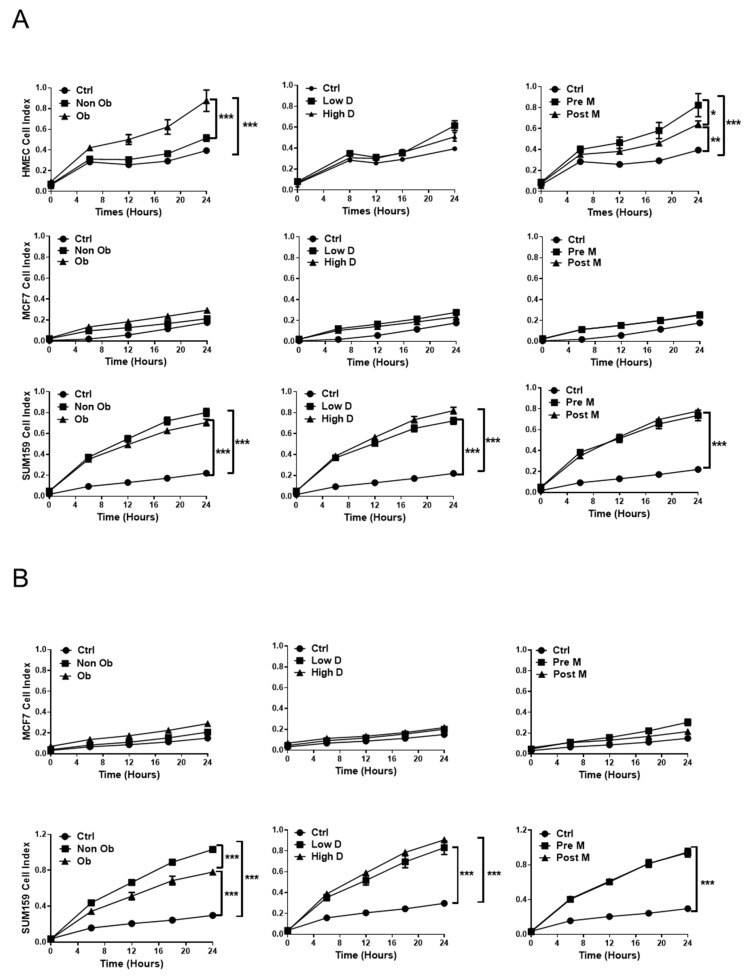
The influence of adipocyte-derived conditioned medium on the migration and invasion of breast cells. Dynamic real-time monitoring of the migration (**A**) or invasion (**B**) of HMEC, MCF7 and SUM159 cells towards either control media or the indicated ACM from ASCs differentiated into adipocytes. Results are expressed as mean ± SEM for three individuals from three independent experiments. * *p* < 0.05; ** *p* < 0.01; *** *p* < 0.001.

Next, the migratory and invasive capacity of normal and malignant breast cells was analyzed (Figure 4). All ACMs had some effect on the migration of HMEC cells, which was particularly marked for obese vs. non obese ACM and Pre-M vs. Post-M ACM (Figure 4A). Unexpectedly, we observed no significant effect on cell migration for MCF-7 cells, independent of which type of ACM was used, whereas all types of ACM strongly increased the migration of SUM159 cells (Figure 4A). Mammary density does not seem to have a major influence on the migration process for any of the cell lines studied (Figure 4A).

HMEC cells were unable to invade under any condition. For MCF-7 cells, the presence of ACM had a marginal, if any, effect on the invasion (Figure 4B). In clear contrast, all ACMs clearly increased the invasive capacity of SUM159 cells, which was most prominent for ACM derived from the tissue of non-obese vs. obese individuals (Figure 4B). Together, these results show that normal breast cells are not able to invade under any conditions, while the ACM has a limited effect on the migration and invasion of MCF-7 cells. In clear contrast, the more aggressive SUM159 cells show increased migratory and invasive capacities in the presence of all types of ACM.

### 2.4. Adipocyte Conditioned Medium Increases the Presence of Lipid Droplets in Breast Cancer Cells

**Figure 5:**
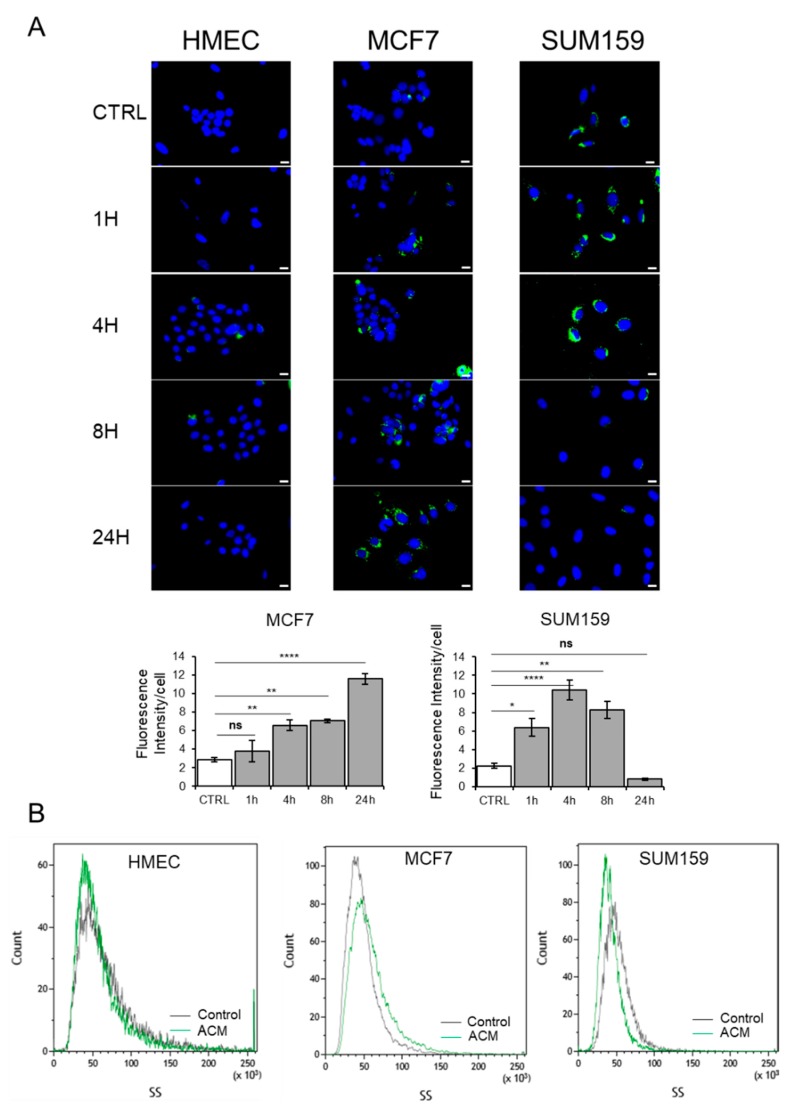
Adipocyte-conditioned medium increases lipids droplets in breast tumor cells. (**A**) Representative images of HMEC, MCF7 and SUM159 cells cultured with control medium or ACM from ASCs differentiated into adipocytes for the indicated times, followed by Bodipy staining. Scale bars, 10 µm (upper panels). The total lipid droplet area was calculated with ImageJ (lower panels). (**B**) Representative side scatter of HMEC, MCF7 and SUM159 cells cultured with control media or with ACM for 24 h. n.s. *p* ≥ 0.05; * *p* < 0.05; ** *p* < 0.01; **** *p* < 0.0001.

The influence of the ACM on proliferation, migration and invasion might be mediated by metabolic reprogramming. We determined if the ACM promoted the increased accumulation of lipids in the tumor cells. Since mammary density had no detectable effect on the proliferation, migration and invasion of tumor cells, we focused on ACM derived from non-obese premenopausal or obese postmenopausal women. Bodipy staining revealed no influence on the lipid accumulation of HMEC cells when incubated with ACM derived from non-obese premenopausal women and Pre-M groups (Figure 5).

In contrast, the corresponding ACM induced a time-dependent increase in cytoplasmic lipid accumulation in MCF7 cells. In comparison, lipid accumulation reached a maximum after 4–8 h, with the SUM159 cells dropping to baseline levels by 24 h (Figure 5A). In agreement with this, we detected elevated levels of cytoplasmic granularity in MCF7 cells and a decrease in the number of granular structures in the cytoplasm of SUM159 cells by 24 h as determined by flow cytometry side scatter analysis (Figure 5B). In comparison, no detectable changes were observed for HMEC cells (Figure 5B). Similar results were obtained when cells were incubated with conditioned medium from obese postmenopausal women). Altogether, these data suggest that MCF7 cells are able to accumulate lipids, in contrast with SUM159 cells, which only accumulate lipids during the first 4–8 h and probably use them as source of energy.

### 2.5. ACM Increases CD36 Expression, Facilitates Fatty Acid Uptake, and Promotes Migration and Invasion of Breast Cancer Cells

**Figure 6:**
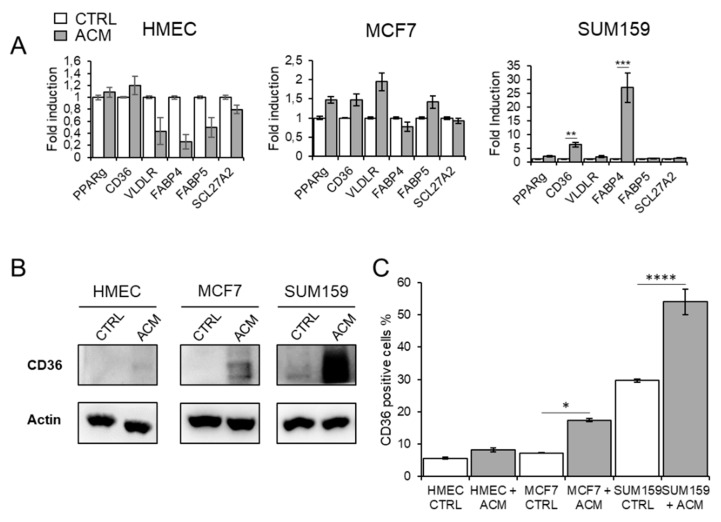

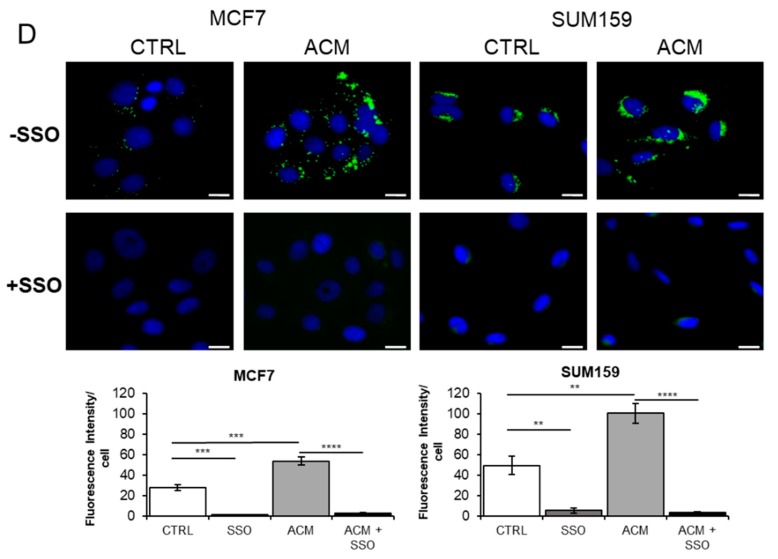
CD36 inhibition reduces adipocyte-induced fatty acid uptake. (**A**) The expression of FA-binding proteins and transporters was determined by qRT-PCR. Fold induction is calculated as the expression of the indicated marker in treated cells compared to untreated controls. Results are expressed as mean ± SEM for three individuals from three independent experiments. ** *p* < 0.01; *** *p* < 0.001. (**B**) Western blot analysis of CD36 were performed for HMEC, MCF7 and SUM159 cells cultured with ACM for 24 h, (**C**) The expression of cell surface CD36 was determined by flow cytometry for HMEC, MCF7 and SUM159 cells cultured with ACM for 24 h. The graph shows the mean ± SEM from three independent experiments. * *p* < 0.05; **** *p* < 0.0001. (**D**) Fatty acid uptake was measured with BODIPY-FA in presence or absence of 150 µM sulfo-N-succinymidyl (SSO). Scale bars, 10 µm (upper panels). Changes in intracellular fatty acid levels are quantified with CellProfiler. The graph shows the mean ± SEM from three independent experiments. ** *p* < 0.01; *** *p* < 0.001; **** *p* < 0.0001. The uncropped blots and molecular weight markers are shown in Supplementary Materials.

To determine whether cancer cells can accumulate FA through an increased uptake of exogenous FA, we studied the effects of ACM from non-obese premenopausal women on the expression of membrane-associated, FA-binding proteins and transporters. We found a modest increase in the expression of *PPARγ*, *CD36* and *VLDLR* mRNA in the MCF7 cells, and a strong increase in the expression of *CD36* and *FABP4* mRNA for the SUM159 cells (Figure 6A). We did not detect any increase in all tested genes in HMEC cells. These data suggest that the ACM promotes upregulation of the scavenger receptor CD36 in the tumor cells, thereby leading to activation of the nuclear receptor PPARγ, which, in turn, results in the transcriptional induction of *CD36* and *FABP4* genes [27,28].

We further investigated the expression of the FA transporter CD36 by flow cytometry analysis and Western blot analysis. The results confirm that ACM promotes the upregulation of cell surface-associated CD36 for both tumor cell lines but not in normal mammary cells (Figure 6B and Figure S6). However, while the overall fraction of CD36-positive cells after ACM exposure was higher for SUM159 cells compared to MCF-7 cells (17% vs. 56%, respectively), the fold increase of CD36 induced by the ACM is higher for the MCF-7 cells, due to the high basal levels of CD36-positive SUM159 cells (Figure 6C). In order to determine whether CD36 is directly involved in the increase of lipid droplets, these cells were treated with sulfo-N-succinymidyl oleate (SSO), a specific inhibitor of CD36. This treatment leads to a decrease in lipid droplets following incubation with ACM (Figure 6D), suggesting that CD36 is involved in the uptake of fatty acids. Similar results were obtained when cells were incubated with conditioned medium from obese postmenopausal women (Figure S7).

**Figure 7:**
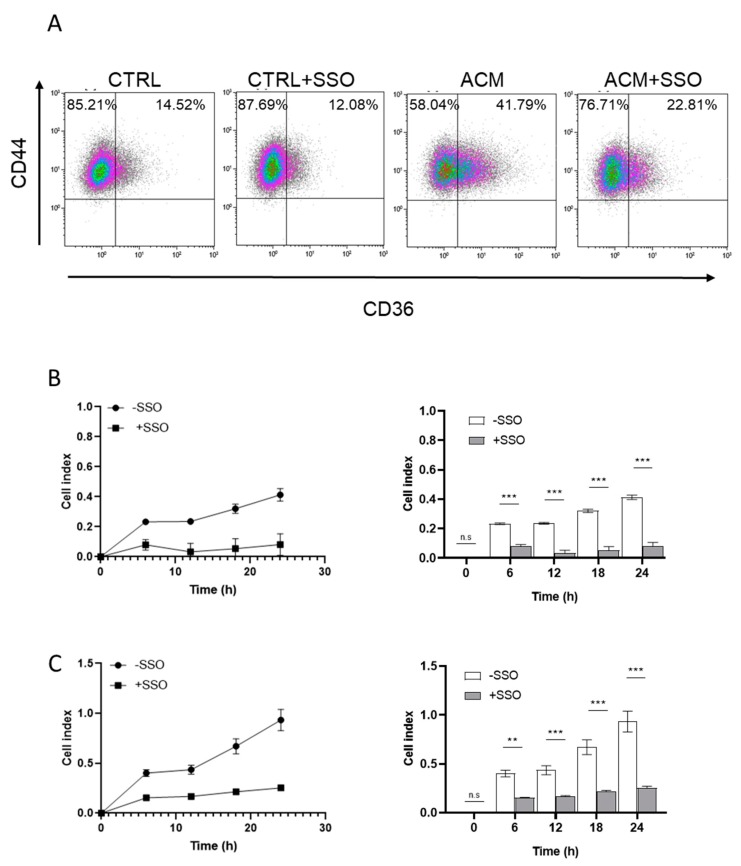
Inhibition of CD36 reduces adipocyte-induced migration and invasion. (**A**) The expression of cell surface CD44 and CD36 were determined by flow cytometry for SUM159 cells cultured with ACM for 24 h in the presence or absence of SSO. (**B**,**C**) Influence of the CD36-specific inhibitor SSO on breast cancer cell migration and invasion. Cancer cells were incubated with ACM in the presence or absence of SSO for 24 h. Dynamic real time monitoring of the migration (B) and invasion (C) of SUM159 cells towards the indicated ACM. Results are expressed as mean ± SEM for three individual samples from three independent. n.s. *p* ≥ 0.05; ** *p* < 0.01; *** *p* < 0.001.

CD44^+^/CD36^+^ cells have been associated with invasion and metastasis in oral carcinoma [29]. We therefore determined if ACM exposure would also increase this phenotype in breast cancer cells. Flow cytometry analysis was used to sort cells based on expression of CD44 and CD36. The results show that exposure to ACM induced a higher fraction of CD44^+^/CD36^+^ cells compared to the untreated control cells (41.8% vs. 14.5%, respectively). Importantly, when SSO was added to the ACM, the CD44^+^/CD36^+^ fraction was reduced from 41.8% to 22.8% (Figure 7A).

To establish if the CD44^+^/CD36^+^ phenotype was associated with an increased invasive potential in the breast cancer cells, cells were treated with ACM in the presence or absence of SSO. Importantly, CD36 inhibition significantly (*p* ≤ 0.001) reduced the ability of the ACM to promote both the migration and invasion of SUM159 cells (Figure 7B,C).

## 3. Discussion

The relationship between adiposity, breast density and breast cancer is complex. Breast adiposity can be influenced by body mass index, menopausal status and mammary density, which are potentially modifiable risk factors for breast cancer. It has been suggested that adipokines, principally secreted by adipocytes of the white AT, are the major contributing factors to breast cancer risk and tumor progression [30]. Circulating adipokine levels are significantly modified by BMI and the menopausal status of healthy women [13,31,32]. We here show that (i), the BMI, menopausal status or breast density of healthy women do not induce major variations in the local secretion of major adipokines and (ii), that secretome can induce metabolic programming of malignant breast cells, which is mediated by an increase in CD36 expression, resulting in increased uptake and accumulation of free fatty acids in a cell type-dependent manner.

The secretion profile of adipose tissue is principally influenced by its body localization [33,34]. Differential gene expression and secretion has been shown between subcutaneous and visceral AT, and also between subcutaneous AT in different sites [34,35]. Our results provide evidence that, in breast, there is no correlation between excess adiposity (high BMI and low mammary density) and local levels of leptin and adiponectin. This is in contrast to the high concentrations of circulating leptin and reduced levels of adiponectin observed in overweight females due, in part, to a higher proportion of subcutaneous fat. This is consistent with previous secretome analysis of breast cancer-associated adipose tissue showing no significant correlation between BMI and adipokine levels [36]. This could be related to local estrogen metabolism, which is known to regulate the levels of adiponectin, leptin and inflammatory adipokines [37,38,39].

Although breast AT may be considered as a subcutaneous adipose tissue, it presents specific characteristics when compared to other subcutaneous adipose tissues. It differs by cyclic structural changes leading to adipocyte differentiation and dedifferentiation, and also by its constant reciprocal interaction with epithelial cells [18].

Several studies have demonstrated the role of adipocytes in the initiation and promotion of tumor growth [8,40]. Because only a subset of women with excess adiposity develop breast cancer, it is unclear exactly which factors and mechanisms favor the malignant transformation. For this reason, we evaluated the influence of breast adipocyte-conditioned medium from different subtypes of healthy women on cell proliferation, migration and invasion toward normal and tumor breast cell lines. Our results suggest that conditioned media from adipocytes, derived from breasts with low or high adiposity, does not affect the proliferation and invasion of normal breast epithelial cells. This is consistent with a recent study showing a very weak association between BMI and breast cancer risk, and no association between non-dense volume and breast cancer risk [41]. In contrast, proliferation, migration and invasion was clearly increased in breast cancer cells, which was most prominent for the highly invasive SUM159 cells and, to a lesser extent, for the less invasive MCF7 cells, suggesting that breast cancer subtype may influence the cross talk between adipocytes and their adjacent breast cancer cells. These findings are in contrast with results obtained with the conditioned medium from breast cancer-associated adipocytes, showing no differences between breast cancer cell lines [36,42], and also with human abdominal adipocytes [43], or murine preadipocyte cells [44,45]. Since the adipocytes used in our study were derived from cancer-free individuals, were induced to differentiate in vitro and had never been exposed to any tumor-conditioned media, the present findings suggest that normal adipocytes do not have an innate ability to promote tumor initiation, although they are able to promote the migration and invasion of invasive breast cancer cells.

Metabolic reprogramming has been firmly established as a hallmark of cancer [46] and cancer cells frequently exhibit alterations in fatty acid metabolism to sustain growth and proliferation [47]. Here, we report that fatty acids are transported into breast cancer cells, but not into normal breast cells. Adipocyte conditioned medium induces the expression of CD36, an integral membrane fatty acid receptor which subsequently promotes the uptake of fatty acids by cancer cells. These findings are in accordance with a recent report showing fatty acid uptake by CD36 in oral carcinoma [29], ovarian cancer [48], and hepatocellular carcinoma [49]. Interestingly, the most aggressive cell line, SUM159, expresses high basal levels of CD36, which further increase after incubation with adipocyte-conditioned medium. The SUM159 cell line also expresses high levels of CD44 [50] and displays a high CD44^+^/CD36^+^ fraction, which is believed to confer an increased metastatic potential [29]. CD36 has been shown to be an STAT3-activated gene [51] and this is consistent with our results showing an activated STAT3 signaling pathway in the presence of adipocyte-conditioned medium in SUM159 cells. However, we also observed that BMI or mammary density did not influence the degree of cellular FA uptake. Therefore, BMI alone may not be an accurate indicator of fatty acid levels [52].

CD36 is a multi-functional protein that can exhibit a variety of different signaling functions upon binding with different ligands. It was shown that SSO could inhibit FA uptake [53] as well as CD36-dependent FA signaling [54]. In the context of invasion, it has been reported that the inhibition of CD36 suppressed the epithelial–mesenchymal transition (EMT) and inhibited the Wnt/β-catenin and TGF-β signaling pathways [49]. This is fully coherent with our study, since SSO treatment was accompanied by decreased levels of lipid droplets, and attenuated migration and invasion underlying the key role of CD36, not only in fatty acids uptake but also in the malignant phenotype.

Taken together, our results show that breast-associated adipocytes promote the aggressiveness of breast cancer cells, which is, at least in part, mediated via increased expression of the CD36 fatty acid receptor.

## 4. Materials and Methods

### 4.1. Tissue Collection

Breast adipose tissues were collected from reduction mammoplasty from donors with no cancer history in accordance with the ethical standards of the local ethical committee. All subjects gave their informed consent to participate in the study (Table S1), and investigations were conducted in accordance with the Declaration of Helsinki, as revised in 2013. The study was approved by the French regulatory authorities (CPP Ile de France 1-2015 mars-DAP 18). According to World Health Organization (WHO), non-obese corresponds to BMI < 30 and obese to BMI ≥ 30. Menopausal status was defined as post-menopausal if women had one year of amenorrhea and the pre-menopausal group could include women in perimenopause state [55]. Mammary density was determined according to the percentage of dense tissue in breast [56].

### 4.2. Cell Lines and Reagent

The human breast cancer cell, MCF-7, obtained from American Type Culture Collection (ATCC, LGC Standards, Molsheim, France), was maintained in DMEM medium (Dulbecco modified Eagle’s medium) supplemented with 10% (*v/v*) FBS (fetal bovine serum), while the SUM159PT cell line (provided by Philippe Benaroch, Cellular transport and immunity team, Curie Institute, Paris, France), was maintained in Ham’s F12 medium supplemented with 5% heat inactivated FBS, 10 mM HEPES, 1 µg/mL hydrocortisone and 5 µg/mL of human insulin. The hTert-immortalized HMEC cell line (provided by Anne-Pierre Morel, EMT and Cancer Cell Plasticity team, Léon Bérard Center, Lyon, France) was maintained in DMEM/F12 medium supplemented with 10% FBS, 10 ng/mL hEGF (human epidermal growth factor), 0.5 µg/mL hydrocortisone, 10 µg/mL insulin, 0.5 µg/mL puromycin. All cell lines were maintained at 37 °C in a humidified atmosphere with 5% CO_2_.

All reagents were purchased from the indicated manufacturers and prepared according to manufacturer’s instructions and used at the following concentrations: Oil Red O: 0.6% (Sigma-Aldrich, Saint-Louis, MO, USA), SSO: 150µM (AbCam, Cambridge, UK), BODIPY 493/503 (Thermofischer, Waltham, MA, USA), 1 µM for microscopy imaging and 10 nM for flow cytometry, BODIPY-FL C12 (Thermofischer), 20 µM, DAPI (Euromedex, Souffelweyersheim, France), 1 µM.

### 4.3. Adipose-Derived Stem Cell Isolation, Adipocyte Differentiation and Collection of Conditioned Media

Blood vessels and glandular tissue were macroscopically removed and the adipose tissue was then minced and dissociated in DMEM supplemented with 2% BSA, 15 mM HEPES pH 7.4, 1 mg/mL collagenase A, 1% penicillin-streptomycin, and 5 µg/mL plasmocin in a shaking warm water bath at 37 °C for 1 h. After full dissociation, the solution was filtered to remove non-digested glandular tissue and centrifuged for 5 min at 1000 rpm. The upper layer containing mature adipocytes and lipids was discarded and the remaining stromal vascular fraction (SVF) was passed through 70 µm nylon filter and centrifuged for 5 min at 1500 rpm. After removal of the supernatant, pelleted ASC was suspended in αMEM (Minimum Eagle’s Medium), supplemented with 10% FBS, 1% penicillin/streptomycin and 2.5 ng/mL basic FGF (fibroblast growth factor) extemporaneously. Cells were differentiated into adipocytes by culturing them in adipogenic induction medium for four days in DMEM high glucose supplemented with 10% Fetal Bovine Serum (FBS) 2 mmol/L glutamine, penicillin/streptomycin (All from Gibco, Invitrogen Corporation, San Diego, CA, USA), 1 µM dexamethasone, 250 µM isobutylmethylxanthine (IBMX), 1 µM insulin and 1 µM rosiglitazone (all from Sigma-Aldrich, Saint-Louis, MO, USA), followed by an adipogenic maintenance medium (1 µM insulin and 1 µM rosiglitazone in DMEM high glucose and 10% FBS). After 14 days, adipogenic differentiation was evaluated by measuring the lipid accumulation using Oil-red-O (Sigma-Aldrich) as described previously [57,58]. Lipid droplet analysis was performed on fixed, Oil Red O- and DAPI-stained cells with cellSens Dimension v1.16 (Shinjuku, Tokyo, Japan). At least 90 cells were analyzed per condition. Conditioned media (ACM) were collected after 24 h of culture in serum-free medium after 13 days of differentiation.

### 4.4. Western Blot and ELISA

Cell extracts were obtained after lysis with RIPA buffer (0.5% sodium deoxycholate, 50 mM Tris-HCl; pH 8, 150 mM NaCl, 1% NP40, 0.1% SDS) supplemented with protease and a phosphatase inhibitor cocktail (Roche, Bäsel, Switzerland) and equal amounts of protein were loaded onto SDS-PAGE gels. After transfer onto nitrocellulose membrane, blots were incubated overnight at 4 °C with the appropriate antibody, followed by incubation with a horseradish peroxidase-conjugated secondary antibody (1/2000, Cell Signaling, Danvers, MA, USA). Bands were visualized using the Clarity™ Western ECL substrate (BIO-RAD, Hercules, CA, USA) on Chemidoc systems [58]. Protein expression was quantified by densitometric analysis of the immunoblots using Image Lab software developed by Bio-Rad. Adipocyte differentiation markers were analyzed using antibodies against FABP4 (Abcam, Cambridge, MA, USA), and peroxisome proliferator-activated receptor γ (PPARγ, Santa Cruz Biotechnology, Heidelberg, Germany). Antibodies directed against STAT3, P-STAT3, AKT, P-AKT, ERK, P-ERK and Vinculin were purchased from Cell Signaling Technology (Danvers, MA, USA), were as the anti-actin-HRP antibody was obtained from Santa Cruz Biotechnology. Leptin, adiponectin, IL6, MCP1 concentrations in the conditioned media were determined by using multi-analyte cartridge ELLA™ immunoassay according to the manufacturer’s instructions (Bio-Techne, San Jose, CA, USA).

### 4.5. Cell Migration, Invasion And Proliferation

This was performed in a continuous non-labeling method using xCELLigence real-time cell analysis (RTCA) technology (Roche, Basel, Switzerland). For the proliferation assay, exponentially growing cells were seeded in an E plate with control medium or adipocyte conditioned medium supplemented with 0.25% SVF. Cell proliferation was assessed for 72 h. All data were recorded and analyzed by the TRCA software. The cell index (CI) value, which is directly influenced by cell spreading and/or proliferation, was used to measure the change in the electrical impedance divided by the background value. For cell migration and invasion, cells in serum-free medium were added to the upper well of the CIM plate, either uncoated (migration) or coated with a thin layer of Matrigel (Corning 60 µL/mL) basement membrane matrix. Control serum-free media or adipocyte-conditioned media were used as chemoattractants in the lower chambers. The plates were incubated for 24 h, and the impedance value measured corresponds to the cell’s passage through the porous membrane, and was expressed as the cell index (CI), divided by the background value.

### 4.6. Flow Cytometry Analysis

Cells cultured in control or adipose-conditioned medium (ACM) supplemented with 2% serum for 48 h were detached with accutase treatment and re-suspended in PBS supplemented with 0.5% serum. Cells were then stained with fluorochrome-conjugated monoclonal antibodies against human CD36-APC, as well as the appropriate isotypic controls (Beckman Coulter) at room temperature in the dark for 20 min. Cells were then washed with PBS containing 0.5% serum and then analyzed by flow cytometry. For Bodipy labeling, cells were cultured in control, or ACM supplemented with 2% serum for 24 h, then detached with accutase treatment and re-suspended in PBS supplemented with 0.5% serum. Aliquots of 1 × 10^6^ cells were fixed with 4% paraformaldehyde for 5 min at room temperature, washed three times with PBS, and incubated in the dark with 10 nM Bodipy 493/503 (Thermofischer) for 5 min at room temperature. After two additional washes with PBS, the cells were analyzed by flow cytometry. The labeled cells were analyzed on an FACS Gallios (Beckman Coulter, Brea, CA, USA) and data analysis was performed using Kaluza software v1.5 (Beckman-Coulter).

### 4.7. Cytoplasmic Lipid Droplet Staining

Cells were cultured in control or adipose-conditioned medium supplemented with 2% serum for various amounts of time, and then were fixed with 4% paraformaldehyde for 10 min at room temperature. After three washes with PBS, the cells were incubated in the dark with 1 µM of Bodipy 493/503 (Thermofischer) and 4′,6′-diamidino-2-phenylindole (DAPI, 1 μg/mL) for 30 min at room temperature. The cells were subsequently visualized by a fluorescent microscope (Evos FL, Thermofischer) with objective ×40.

### 4.8. Fatty Acid Uptake

Cells were cultured in control or adipose-conditioned medium supplemented with 2% serum for 16 h. A 150 µM solution of sulfosuccinimidyl Oleate (SSO; Abcam) was added to the medium to inhibit lipid uptake for 15 min. After washes, 20 µM solution of Bodipy-dodecanoic acid fluorescent FA analogue (BODIPY 558/568 C12) were added for 20 min. The cells were subsequently visualized by a fluorescent microscope (Evos FL) with objective ×40.

### 4.9. Quantitative Real Time RT-PCR

Reverse transcription-PCR (RT-PCR) analysis was carried out as described previously [59]. Gene expression was normalized to β-actin and RPLP0 (also known as 36B4). The sequence of primers used is indicated in Table S2.

### 4.10. Adipokine Array

Serum-free conditioned media was applied to an adipokine antibody array membrane (R&D systems, Minneapolis, MN, USA), in accordance with the manufacturer’s instructions, to detect relative levels of 58 adipokines. Images were acquired on Chemidoc system (BIO-RAD). Image Lab^TM^ software v6.0.1 (BIO-RAD) was used for the densitometric analysis.

### 4.11. Fatty Acid Methyl Ester Analysis

A micro-method for total lipid extraction and methylation was applied from 500 μL of the cell supernatant. Fatty acid methyl esters (FAME) were prepared and assayed by GC-MS in the positive chemical ionization mode with ammonia as the reagent gas (GC6890-MS5975; Agilent Technologies, Les Ulis, France) as described previously [60]. The response factors of fatty acids were calibrated with a weighed mixture (FAME Mix Supelco^®^ 37, Sigma-Aldrich Chimie, L’Isle d’Abeau Chesnes, Saint Quentin-Fallavier, France).

### 4.12. Statistical Analysis

Data were expressed as mean ± SEM. Statistical analysis was performed using t-test or the one-way ANOVA test with GraphPad Prism 6 v6.01 (GraphPad Software, Inc., San Diego, CA, USA).

## 5. Conclusions

In conclusion, we here show that free fatty acids released by breast adipocytes profoundly influence the proliferation, migration and invasiveness of malignant breast cells, but not normal breast epithelial cells, independently of body mass index, menopausal status and mammary density. We further show that the increased migration and invasion is, at least in part, mediated by increased expression of the fatty acid receptor CD36, thereby facilitating exogenous fatty acid uptake. These findings suggest that CD36 targeting may be useful to attenuate breast cancer metastasis.

## Supplementary Materials

The following are available online at https://www.mdpi.com/2072-6694/11/12/2012/s1, Figure S1: The whole blot, showing all the bands with all molecular weight markers on the Western blotting, Figure S2: Fatty acids profiles of ASCs-conditioned medium, Figure S3: Bar chart representing the proliferation of cells treated with control or ASCs-conditioned medium at 12, 24, 48 and 72 h, Figure S4: Western blot quantification, Figure S5: The whole blot showing all the bands with all molecular weight markers on the western blotting, Figure S6: The whole blot showing all the bands with all molecular weight markers and Western blot quantification, Figure S7: CD36 inhibition reduces adipocyte-induced fatty acid uptake, Table S1: Characteristics of the women, Table S2: list of primers.
