# Supplementary material for: Breast-Associated Adipocytes Secretome Induce Fatty Acid Uptake and Invasiveness in Breast Cancer Cells via CD36 Independently of Body Mass Index, Menopausal Status and Mammary Density
Source: Cancers (Basel). 2019 Dec 13;11(12):2012. doi: 10.3390/cancers11122012 (PMC6966437; doi:10.3390/cancers11122012)
Supplement: Supplementary file 1 [file cancers-11-02012-s001.pdf]

Supplementary Materials: Breast-Associated Adipocytes Secretome Induce Fatty Acid Uptake and Invasiveness in Breast Cancer Cells via CD36 Independently of Body Mass Index, Menopausal Status and Mammary Density

Maurice Zaoui, Mehdi Morel, Nathalie Ferrand, Soraya Fellahi, Jean-Philippe Bastard, Antonin Lamazière, Annette Kragh Larsen, Véronique Béréziat, Michael Atlan and Michèle Sabbah

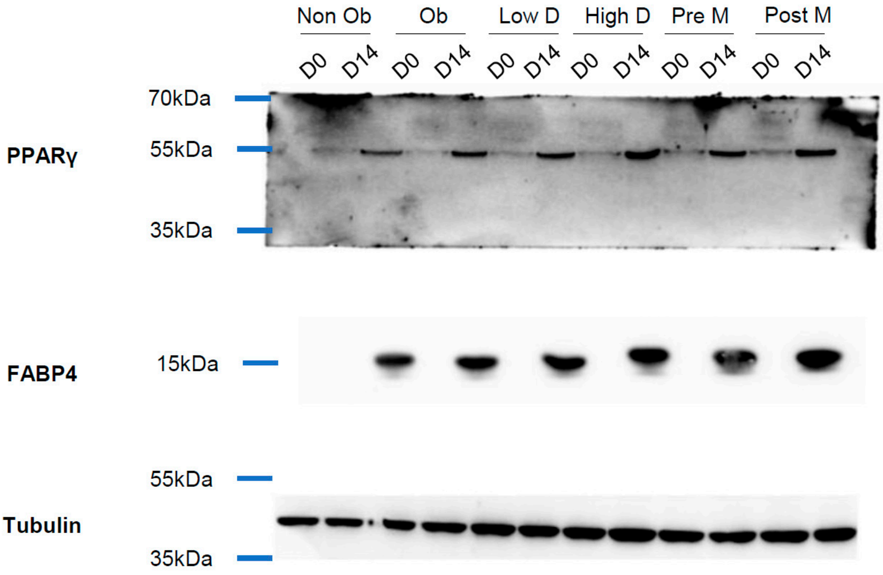

	Non Ob		Ob		Low D		High C		Pre M		Post M	
	D0	D14	D0	D14	D0	D14	D0	D14	D0	D14	D0	D14
PPARγ	0.02357644	0.55473104	0.00570736	0.51658066	0.00999297	0.54375981	0.0170117	0.5330375	0.00895798	0.52812307	0.02763506	0.55649261
FABP4	0.00944453	1.82557544	0.01444163	1.94452062	0.01174121	1.92930307	0.010490924	1.78892917	0.01450824	1.92877003	0.00684606	1.78972872
Tubulin	3847340.96	4079989.33	3115301.67	4831050	3718620.44	4617883.33	3860917.568	4024209	3009060.16	4556796	4372722.87	4115840

Figure S1. The whole blot showing all the bands with all molecular weight markers on the western blotting.

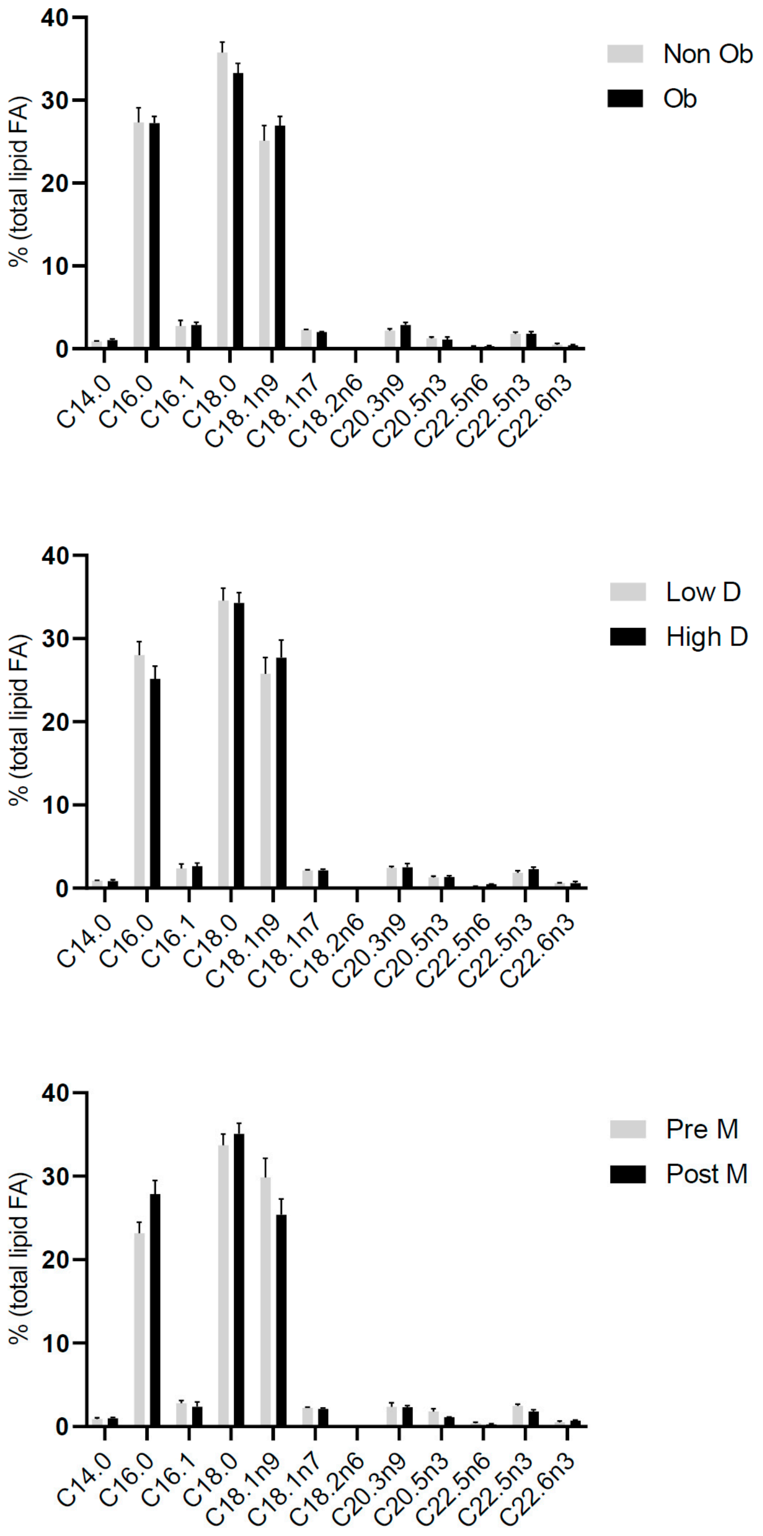

Figure S2. Fatty acids profiles of ASCs-conditioned medium.

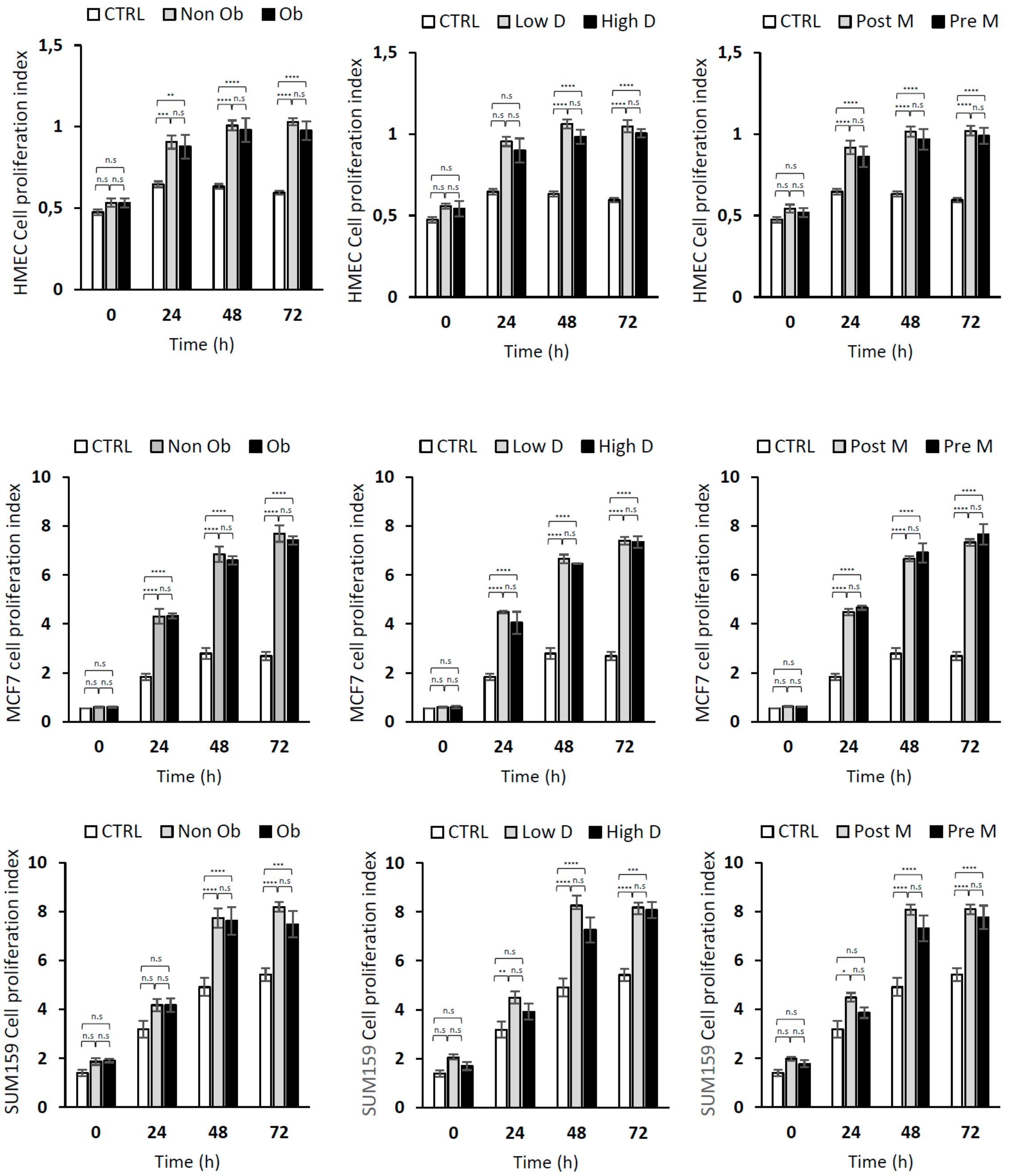

Figure S3. Bar chart representing the proliferation of cells treated with control or ASCs-conditioned medium at 12, 24, 48 and 72 h. n.s. no significance; $p \geq 0.05$; ** $p < 0.01$; **** $p < 0.0001$.

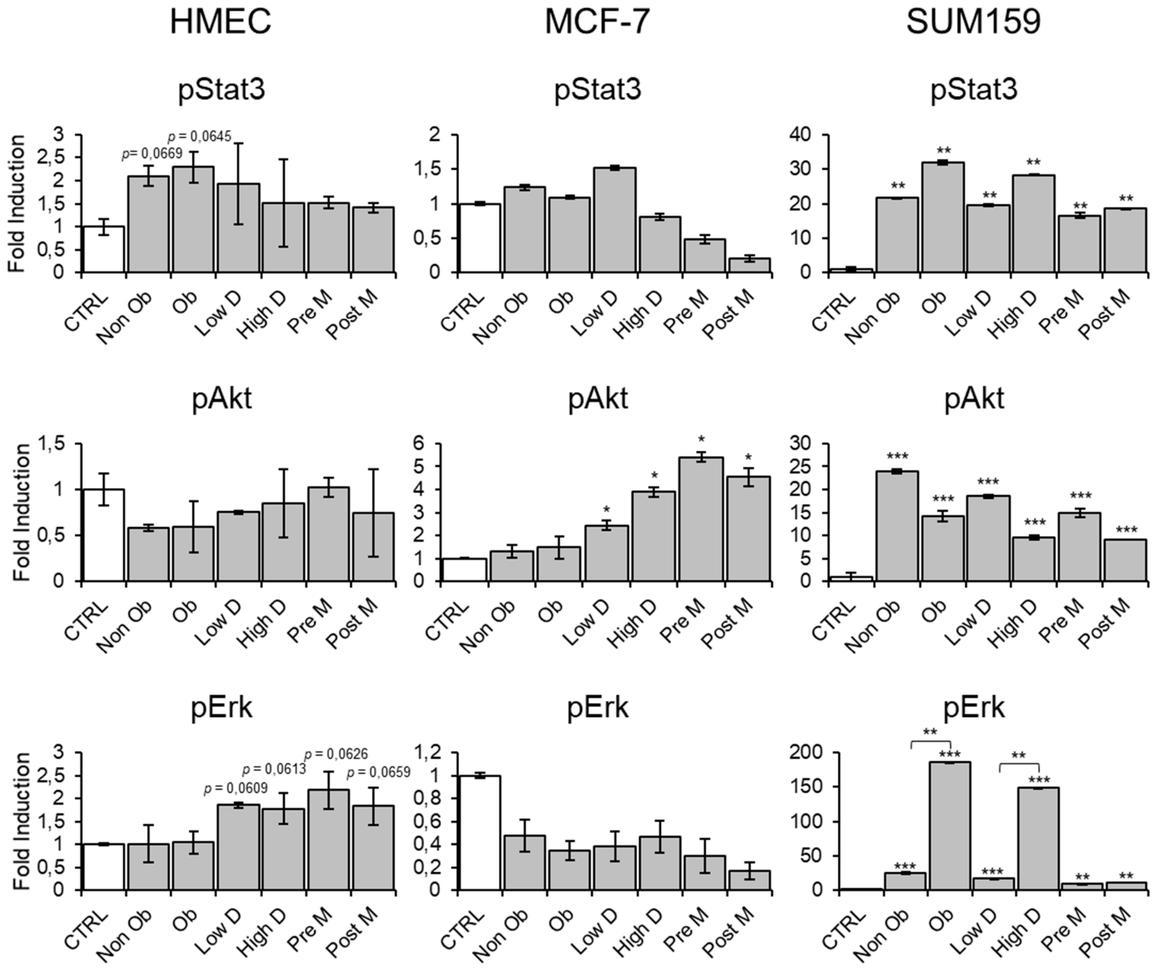

Figure S4. Western blot quantification. n.s. $p \geq 0.05$; * $p < 0.05$; ** $p < 0.01$; *** $p < 0.001$.

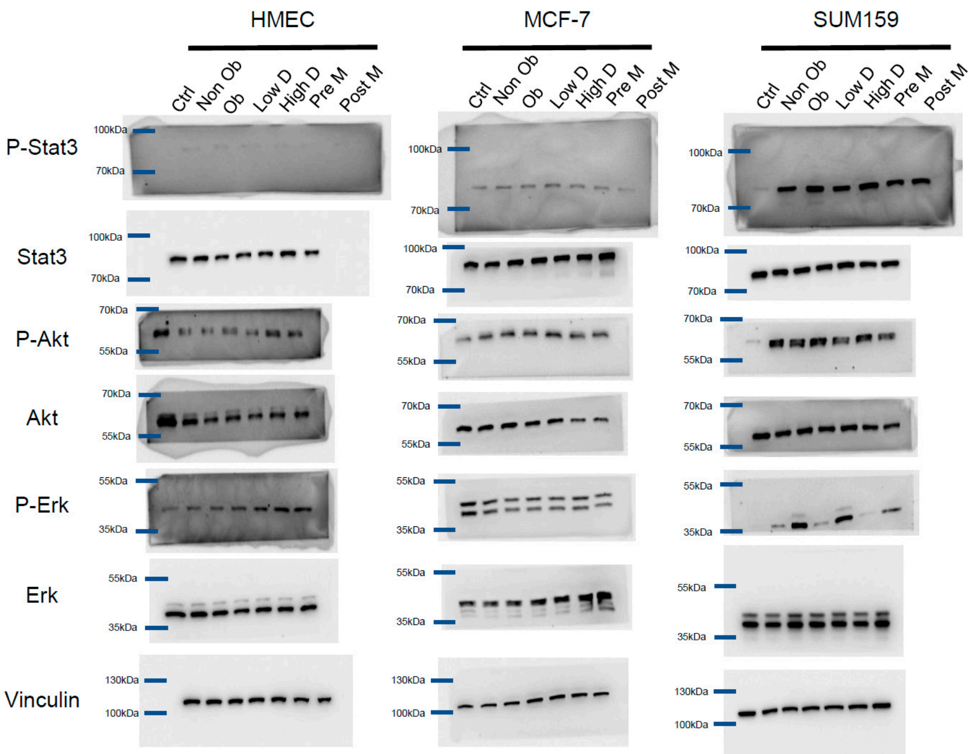

	CTRL	Non Ob	Ob	Low D	High D	Pre M	Post M
**HMEC**				**P-Stat3**			
	15227,833	197199,83	136671,2	153458	78324,167	53004,048	58294,774
				**Stat3**			
	2959536,2	3308379,4	2523184,7	3467647,7	3207564,9	3003339,3	3556861,8
				**P-Akt**			
	3024567,6	1042801	828037,31	1211626,7	848201,38	1913805,3	1466494,8
				**Akt**			
	8526431,4	3769415,5	2219260,3	2823036,4	1994015,3	2552461,1	2555231,2
				**P-Erk**			
	475732,21	635490,81	684285,7	914985,13	951181,26	1621092,7	1572491,9
				**Erk**			
	5629621,2	5551177,1	4391582	3627586,4	4484970,4	4272323,3	4669436,3
				**Vinculin**			
	4438654	4302443,7	4063860,6	3858391,9	3964239,7	3106775,8	3083843,1
	**CTRL**	**Non Ob**	**Ob**	**Low D**	**High D**	**Pre M**	**Post M**
**MCF7**				**P-Stat3**			
	440612,67	580652	506566,33	715994,33	474114,33	430913,67	211705,67
				**Stat3**			
	2581686,2	2990407,9	3555990,6	3568760,5	4456367,6	5061525,7	6783989,9
				**P-Akt**			
	1037372,6	1835882,8	2159373,7	2177195,4	2350641,3	1834723,7	1860548
				**Akt**			
	3911123,6	3497833,8	4132185,1	3125838,5	2961206,7	1524238,8	1618868,5
				**P-Erk**			
	11206742	6649025,9	4879770,2	5670741	5628552,8	6671086,8	5056920,1
				**Erk**			
	3176037,4	2597719,9	3021515,1	3936164,5	4424711,8	7490048,6	8746421,6
				**Vinculin**			
	1975132	1850752,7	2331138,3	3263971	3380255,3	2913344,3	2389018,7
	**CTRL**	**Non Ob**	**Ob**	**Low D**	**High D**	**Pre M**	**Post M**
**SUM159**				**P-Stat3**			
	133709,61	2935036,6	5300257,2	3431144,2	5154562,6	2975151,6	3358349,6
				**Stat3**			
	5450142,1	4924503,2	5924335,3	6032831,2	6482056,9	5973105	4971467,3
				**P-Akt**			
	285208,42	4495969,2	3784071,2	4572961,8	2788582	4108692,8	2764441,7
				**Akt**			
	5368697,1	3530568,3	4350263,2	3776721,3	4268788,5	3104549,3	2573428,3
				**P-Erk**			
	50603,12	679063,32	5666001,9	433907,52	4491540	104873,64	1561489
				**Erk**			
	461485,15	238580,68	241137,17	187699,66	214817,47	61566,396	561513,62
				**Vinculin**			
	5904084,3	3676117,9	3588657	3968933,4	3891803	4513584,3	6180057,4

Figure S5. The whole blot showing all the bands with all molecular weight markers on the western blotting.

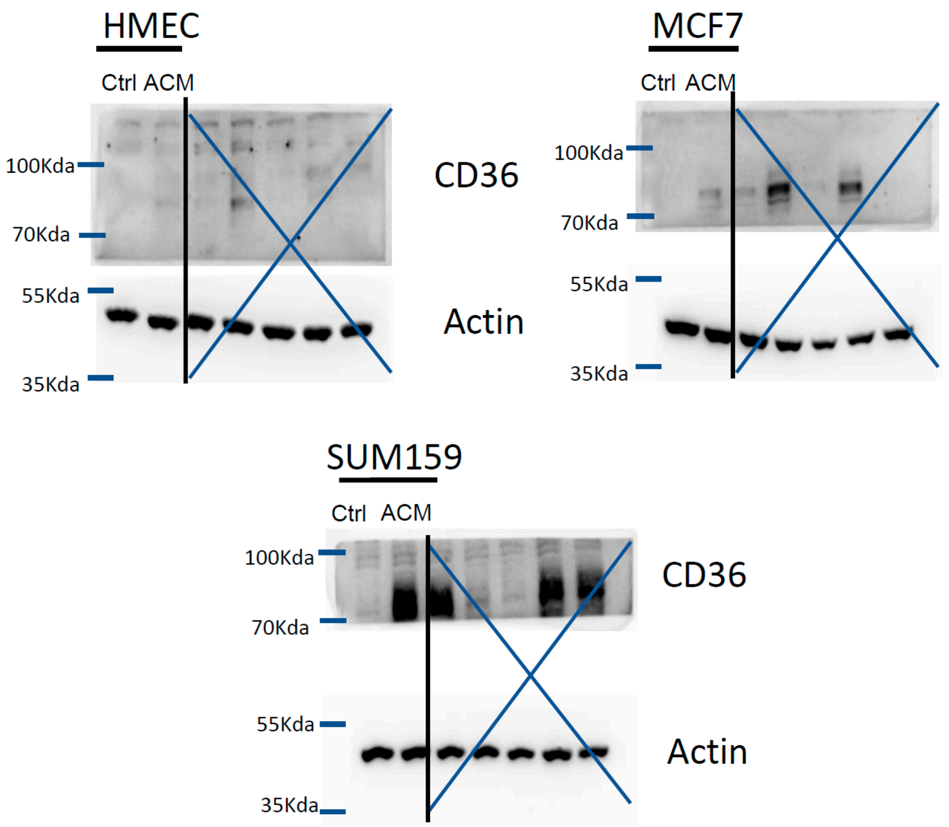

	HMEC		MCF7		SUM159	
	Ctrl	ACM	Ctrl	ACM	Ctrl	ACM
CD36	29192,3	41303,8	95968,5	995057,8	62149,9	18133062,3
Actin	12818881,3	12473414,3	13731232,3	11296635,5	13259668,3	11323904,7

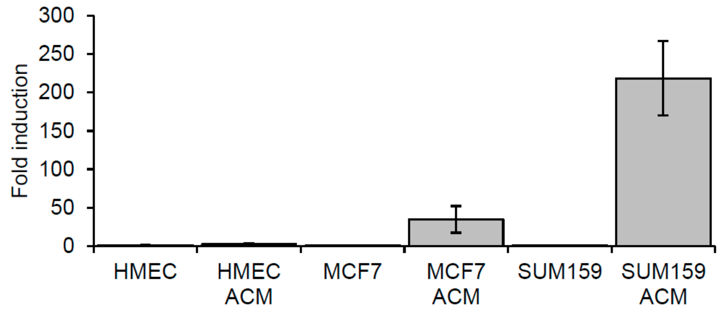

Figure S6. The whole blot showing all the bands with all molecular weight markers and western blot quantification.

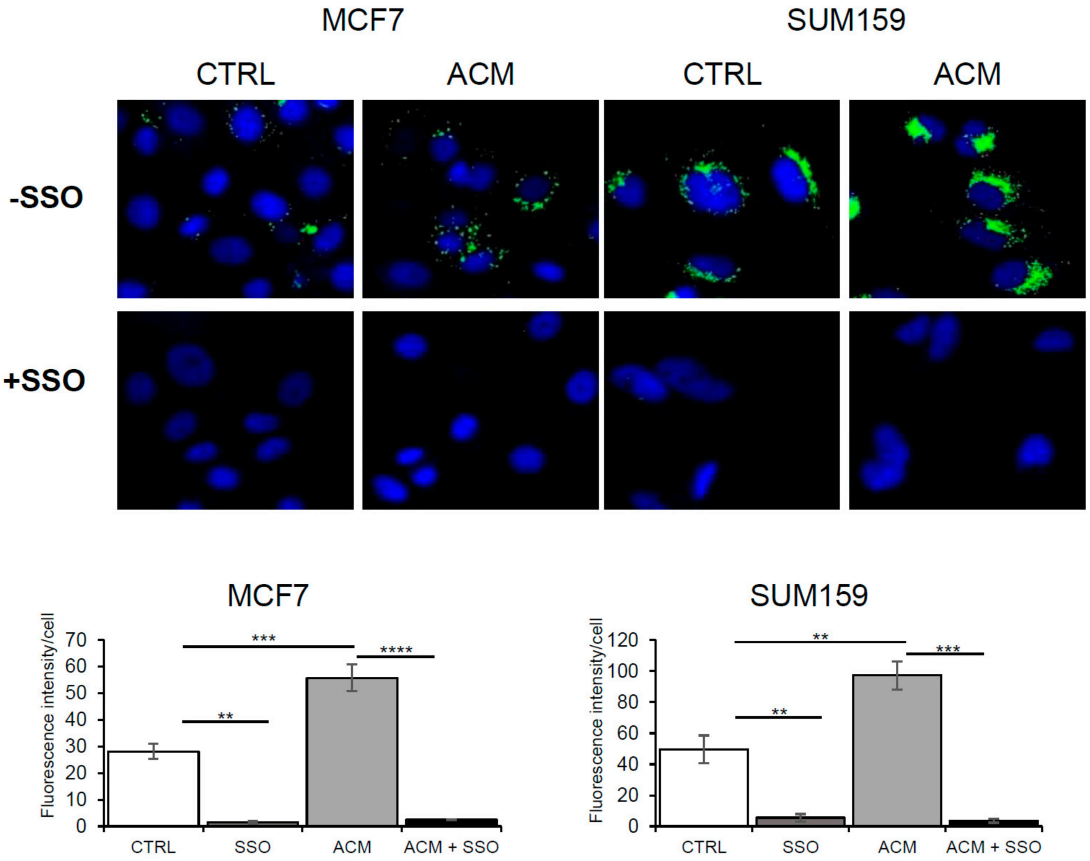

Figure S7. CD36 inhibition reduces adipocyte-induced fatty acid uptake.

Table S1. Characteristics of the women.

Patient	Age	Height (m)	Weight (kg)	BMI (kg/m²)	Mammary Density	Menopausal Status
**Patient 1**	35	1.62	88	33.5	Low D	−
**Patient 2**	43	1.6	71	27.7	Low D	−
**Patient 3**	54	1.55	53	22.1	Low D	+
**Patient 4**	61	1.58	65	26	High D	+
**Patient 5**	56	1.6	76	30	Low D	+
**Patient 6**	62	1.63	70	26.3	Low D	+
**Patient 7**	16	1.63	63	23.7	High D	−
**Patient 8**	50	1.72	78	26.4	High D	+
**Patient 9**	18	1.75	105	34.3	Low D	−
**Patient 10**	60	1.62	91	34.7	Low D	+
**Patient 11**	51	1.65	72	26.4	High D	+
**Patient 12**	51	1.72	78	26.4	Low D	+
**Patient 13**	55	1.63	80	30.1	High D	+
**Patient 14**	17	1.7	80	27.7	High D	−
**Patient 15**	18	1.68	83	30	Low D	−
**Patient 16**	44	1.77	71	22.7	High D	−

Table S2. list of primers.

Gene	Forward	Reverse
**D36**	TGGTACAGATGCAGCCTCAT	AGGCCTTGGATGGAAGAACA
**FABP4**	GGGCCAGGAATTTGACGAAG	ACTTCAGTCCAGGTCAACGT
**FABP5**	GCTGAACCAATGCACCATCT	AGGAGTGGGAATAGCTTTGCG
**PPARγ**	TTGCAGTGGGGATGTCTCAT	TTTCCTGTCAAGATCGCCCT
**SLC27A2**	ACCACAGGTCTTCCAAAAGCA	AGTCCGCAAGGCAAGAGTAG
**VLDLR**	GAATGTGAGGATGGCAGCTG	CAGTCCTGCTCCTGGTTACA
**βActine**	GGACTTCG AGCAAGAGATGG	AGCACTGTGTTGGCGTACAG
**36B4**	GGACTTCG AGCAAGAGATGG	AGCACTGTGTTGGCGTACAG

© 2019 by the authors. Licensee MDPI, Basel, Switzerland. This article is an open access article distributed under the terms and conditions of the Creative Commons Attribution (CC BY) license (http://creativecommons.org/licenses/by/4.0/).
